# Stone in the Negro

**Published:** 1861-01

**Authors:** 


STONE IN THE NEGRO.—In a letter to the senior editor, dated Mobile, December 8, 1860, Professor J. C. Nott makes the following remarks upon this subject:—

"I operated to-day on a case of stone in a negro boy, and on referring to your book, it brought to mind the fact, which I had before seen stated by yourself and others, viz., the infrequency of this disease in the negro race. My experience is to the contrary. I have not been very careful in keeping notes of my operations, not expecting to write on the subject, and cannot, therefore, state accurately the number of cases operated on by myself in the twenty-five years I have been in Mobile, but the entire number is about twenty, out of which I can count up eight in negroes, two of which were last winter. The ages have been from three to twenty-five years; most of them have been boys from five to ten years.—While writing, I recollect another case, which is very remarkable. This was a negro woman in the interior of the State, who passed five at one gush after suffering several days very severely; they were sent to me by a country physician; they are about the size of olives, somewhat flattened. I have just measured them, and they are all from three-quarters to seven-eighths of an inch in diameter, and nearly as broad as long. I have heard of at least four or five others among country physicians. All my cases have been brought from counties of South Alabama."

